# Reproductive success of individuals with different fruit production patterns. What does it mean for the predator satiation hypothesis?

**DOI:** 10.1007/s00442-012-2502-x

**Published:** 2012-10-19

**Authors:** Magdalena Żywiec, Jan Holeksa, Mateusz Ledwoń, Piotr Seget

**Affiliations:** 1 Institute of Botany, Polish Academy of Sciences, ul. Lubicz 46, 31-512 Kraków, Poland; 2 Institute of Systematics and Evolution of Animals, Polish Academy of Sciences, ul. Sławkowska 17, 31-016 Kraków, Poland; 3 Department of Biodiversity and Nature Conservation, Agricultural University, Al. 29 Listopada 46, 31-425 Kraków, Poland

**Keywords:** Masting, Pre-dispersal seed predation, Selection, *Sorbus aucuparia*, Synchrony

## Abstract

The predator satiation hypothesis states that synchronous periodic production of seeds is an adaptive strategy evolved to reduce the pressure of seed predators. The seed production pattern is crucial to the predator satiation hypothesis, but there are few studies documenting the success of individuals that are in synchrony and out of synchrony with the whole population. This study is based on long-term data on seed production of *Sorbus aucuparia* and specialised pre-dispersal seed predation by *Argyresthia conjugella*, in a subalpine spruce forest in the Western Carpathians (Poland). At the population level, we tested whether functional and numerical responses of predators to the variation of fruit production operate. At the individual level, we tested whether individuals with higher interannual variability in their own seed crops and higher synchrony with the population have higher percentages of uninfested fruits. The intensity of pre-dispersal seed predation was high (average 70 %; range 19–100 %). There were both functional and numerical responses of predators to the variation of fruit production at the population level. We found that individuals that were expected to be preferred under seed predator pressure had higher reproductive success. With increasing synchrony of fruit production between individual trees and the population, the percentage of infested fruits decreased. There was also a negative relationship between the interannual variation in individual fruit production and the percentage of infested fruits. These results confirm selection for individuals with a masting strategy. However, the population does not seem well adapted to strong seed predation pressure and we suggest that this may be a result of prolonged diapause of *A. conjugella*.

## Introduction

Pre-dispersal seed predation may have important ecological and evolutionary consequences for plants (Hulme and Benkmann 2002; Kolb et al. 2007). For most species, pre-dispersal predation rates are low (Kolb et al. 2007). For some species, however, seed losses due to predation are considerable (Windus and Snow 1993; Crawley and Long 1995; Kelly and Sullivan 1997; Fedriani et al. 2004). In consequence, seed predation can significantly affect the reproductive success of individuals and recruitment in plant populations (Louda 1982; Fenner 1985; Maron et al. 2002; Kolb et al. 2007). If a seed predator differentiates between plants that vary in some heritable traits, it exerts selective pressure that favours one of them (reviewed in Kolb et al. 2007). To avoid predation, physical and chemical defence mechanisms have evolved in plants, and also specific interannual patterns of seed production (Hulme and Benkmann 2002).

Masting, the synchronous highly variable seed production among years by a population of perennial plants, is explained in many species as a reproductive strategy evolved in response to seed predation (Janzen 1971; Silvertown 1980; Kelly 1994; Kelly and Sork 2002). High interannual variation in fruiting is suggested to be an adaptive strategy reducing the pressure of seed predators. The predator satiation hypothesis is based on three essential elements: years of heavy crops to satiate predators by providing the overabundance of food or oviposition sites (functional response of predators), years of low seed production to reduce the predator population density via starvation or reduced reproductive success (numerical response of predators) and, to make the first two elements effective, synchrony among individuals in a population (Silvertown 1980; Kelly and Sork 2002). Although masting assumes missed opportunities for reproduction in some years, stochastic matrix population models confirm its considerable effectiveness in minimising the effects of seed predation and increasing plant fitness (Satake and Bjørnstad 2004; Visser et al. 2011). In nature, many examples of predator satiation have been documented (Shibata et al. 2002; Yasaka et al. 2003; Kon et al. 2005; Sun et al. 2007; Espelta et al. 2008, 2009; Poncet et al. 2009).

The seed production pattern is crucial to the predator satiation hypothesis. Although the masting strategy works at the population level, it is based on the seed production patterns of individual plants. Seed production synchrony among individuals is an important element of this strategy. Satiation is most effective when individuals have heavy crop years synchronously separated by low crop years. At the heart of the predator satiation hypothesis is its adaptive character. Seed production synchrony among individuals seems to enhance the individual fitness of plants. According to the satiation hypothesis, an individual producing seeds out of synchrony would be selected against, as its seeds would not be protected from predation by the effect of satiation (functional response of predators), which safeguards seeds produced synchronously in mast years (Silvertown 1980; Rees et al. 2002).

Seed predators may exert selective pressure on plants, differentially reducing the seed production of plants that differ in heritable traits (Kolb et al. 2007). There are studies showing that seed predation exerts selective pressure on plant morphological traits (Brody 1992; Gómez and Zamora 1994; Fenner et al. 2002; Kolb and Ehrlén 2010) and on the phenology of flowering and fruiting (Augspurger 1981; Klips et al. 2005). However, little attention has been paid to the individual reproductive success of trees in species under strong seed predation (Lalonde and Roitberg 1992). Studies comparing the success of individuals that are in synchrony and out of synchrony with the whole population are among the most revealing (Kelly 1994). Long-term data on the seed production of individuals and the predation they experience are required in order to directly study selective pressure (Kelly and Sork 2002).

Our study is based on long-term data on *Sorbus aucuparia* (rowan) fruit production and on infestation of its fruits by a specialised seed predator, *Argyresthia conjugella* (Lepidoptera, Yponomeutidae). Rowan is a fleshy-fruited tree species reported as having considerable interannual variation of fruit production. As it experiences strong seed predation by *A. conjugella*, masting in this species has been suggested to be an adaptive defence against such predation (Kobro et al. 2003; Satake et al. 2004). In a previous study, we analysed the fruit production pattern of the studied population in 2000–2010. The coefficient of variation of fruit production for the population (CV_*p*_) was 1.07. We found the fruit production pattern to be strongly influenced by weather conditions. However, the rowans responded synchronously only to unfavourable weather conditions; in good years, the fruit production of individual trees varied considerably. Moreover, endogenous cycles of individual fruit production were not found. In consequence, rowan trees represent a range of fruit production patterns (Żywiec et al. 2012). In this study, we determined the level of fruit infestation by *A. conjugella* and tested whether there are functional and numerical responses of predators to the variation of fruit production at the population level. The predator satiation hypothesis predicts a negative correlation of fruit infestation intensity with current-year fruit production (functional response of predators) and with the ratio of current to previous-year fruit crops (numerical response of predators). Under high seed predation, the existence of functional and numerical responses of predators would suggest that the seed predator exerts selective pressure on the population. The presence of several fruit production patterns in the population gives an opportunity to test whether individuals that (1) are better synchronised with the population and (2) show higher variation of fruit production have lower percentages of infested fruits (i.e. higher reproductive success) and finally are better fitted for pre-dispersal seed predation. We asked the following questions:

1. Are there functional and numerical responses of predators to variation of fruit production at the population level?
2. Is the reproductive success of individuals correlated with synchrony of individual fruit production with population fruit production?
3. Is the reproductive success of individuals correlated with the level of variation of fruit production?

## Materials and methods

### Study species

Rowan *Sorbus aucuparia* L. (Rosaceae, Maloideae) is a deciduous fleshy-fruited tree. It lives 100–150 years and reaches 15–20 m in height (Kullman 1986; Hofgaard 1993). Flower buds develop in the year before anthesis (Sperens 1997b), and clusters of hermaphroditic flowers are produced in late spring (May–June) which are pollinated by a wide range of insects (Raspé et al. 2000; Pías and Gutáin 2006). The fruits are subglobose pomes ripening in August–September, and the seeds are dispersed by animals, mainly birds and mammals (Raspé et al. 2000).

The apple fruit moth *Argyresthia conjugella* Zell. is a microlepidopteran specialist seed predator of rowan. The adults lay eggs on newly initiated fruits. The larva feeds on developing seeds and fruit pulp (Sperens 1997a; Kobro et al. 2003). After several weeks, it leaves the fruit to pupate in the ground beneath the tree. Most of the pupae emerge the following summer, but some may not emerge until 2 years later (Sperens 1997a; Kobro et al. 2003).

### Study site

The study was done in a subalpine old-growth spruce forest on the north slope of the Babia Góra massif (1,725 m a.s.l.) in the Western Carpathians (Poland). Tree stands in the studied forest are built of *Picea abies* with sporadic occurrence of *S*. *aucuparia*. Small rowan thickets are a dynamic component of the forest, occurring in spruce stand gaps resulting from bark beetle outbreaks or windstorms (Holeksa et al. 2008; Żywiec 2008; Żywiec and Ledwoń 2008; Żywiec and Holeksa 2012).

### Data collection

Fruit production was studied on a 27-ha (564 × 480 m) rectangular plot representative for subalpine spruce forest, at 1,170–1,310 m a.s.l. on the north slope of the Babia Góra massif. All rowan trees (*n* = 346) were searched for fruits at the beginning of September in 2004–2011. Fruit production was measured by binocular observations, counting the number of infructescences on individual trees. Five infructescences were randomly taken from each tree and all the fruits in them were counted. The fruit production of an individual tree was estimated as the product of the number of infructescences and the average number of fruits in five infructescences (see Żywiec et al. 2012). Annual fruit production at population level was measured as mean fruit production per hectare.

To study pre-dispersal seed predation, 30 individuals were randomly chosen from among the trees bearing fruits in 2005. Over 2005–2011, three infructescences per tree (from different distant branches) were collected at the beginning of September. Ten fruits were taken randomly from each infructescence and dissected for seeds predated by *A. conjugella*. Because there were no remnants of seeds in many fruits, it was not possible to estimate the number of seeds predated. For this reason, we took the percentage of fruits with signs of *A. conjugella* feeding (infested fruits) as the measure of predation at the individual level.

### Data analysis

To determine the effect of functional response of predators to the variation of fruit production, we calculated the Pearson correlation coefficients for the population-level relationship between the percentage of infested fruit and fruit production. To determine whether a numerical response of predators to the variation of fruit production operates, we analysed the correlation of current-year fruit infestation with the ratio of current to previous-year fruit crops. The fruit infestation and fruit production data were log-transformed to obtain normal distributions.

For each tree and each year, we calculated the percentages of infested fruits. The percentage of fruits infested was also calculated for each tree for the combined years of 2005–2011, using sums of fruits produced and fruits infested in these years. As the measure of tree reproductive success, we assumed the percentage of fruits uninfested in 2005–2011.

We wanted to determine whether individual infestation intensity in the 7 years was related to the level of fruit production synchrony between individuals and the population. We used Pearson correlation coefficients as the measure of synchrony between the pattern of individual fruit production and the pattern of population fruit production (Buonaccorsi et al. 2001; Koenig et al. 2003). Then, we used linear regression analysis to find the relationship between individual percentages of infested fruits for the combined years of 2005–2011 and individual level of synchrony.

We also used linear regression analysis to find out whether individual infestation intensity for the 7 years taken together was related to individual variation of fruit production (CV_*i*_). CV_*i*_ was calculated using the standard deviations and means of individual tree fruit production in 2004–2011 (see Herrera et al. 1998; Koenig et al. 2003).

## Results

### Population-level fruit production and infestation

**Fig. 1:**
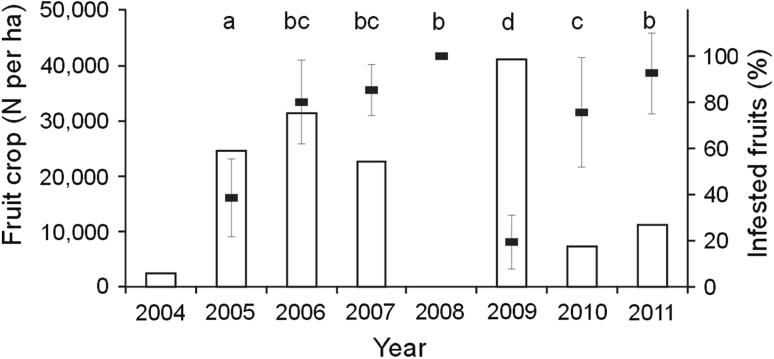
Fruit production by *Sorbus aucuparia* (*bars*) and infested fruits (%) (mean ± SD) by *Argyresthia conjugella* in subalpine spruce forest. *Percentages with different letters* differ significantly (ANOVA: *P* < 0.05)

At the population level, fruit production was heaviest in 2009, reaching 41,154 fruits/ha, and was also heavy in the three consecutive years of 2005–2007 (Fig. 1). The lowest fruit production was in 2008 (12 fruits/ha).

There were significant differences in the percentage of infested fruits between years (ANOVA: *F*_6,169_ = 80,9; *P* < 0.0001; Fig. 1). Fruit infestation was lowest in 2009 (19 %) and 2005 (38 %). It was highest in 2008 (100 %), in the year of the lowest fruit production, but it was also high in 2006 (80 %) and 2007 (85 %), which were years of relatively heavy fruit production. In five of the seven studied years, fruit infestation exceeded 75 %.

**Fig. 2:**
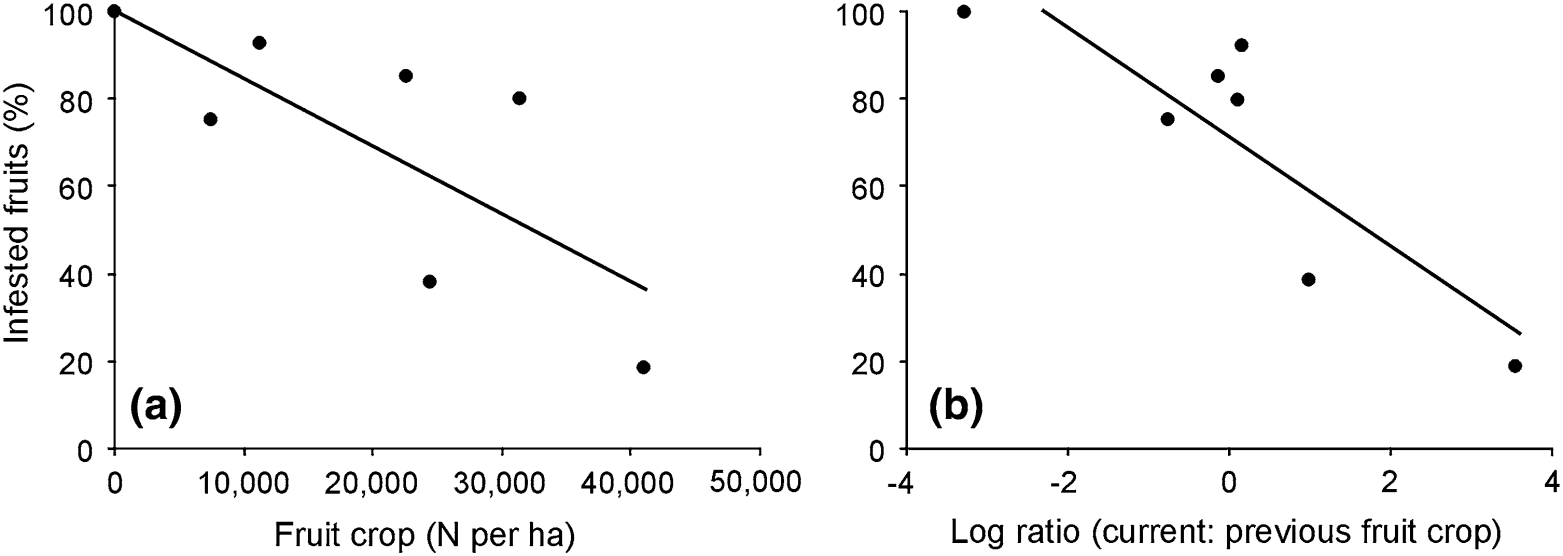
Infested fruits (%) as a function of **a** population fruit crop and **b** log ratio of the current to previous-year fruit crops

Both functional and numerical responses of predators to the variation of fruit production were revealed at the population level. The percentage of infested fruits in the population was related negatively with annual fruit production (*R* = −0.77; *P* = 0.04; Fig. 2a) and with the ratio of the current to previous-year fruit crops (*R* = −0.83; *P* = 0.02; Fig. 2b).

### Fruit infestation and reproductive success of individuals

**Fig. 3:**
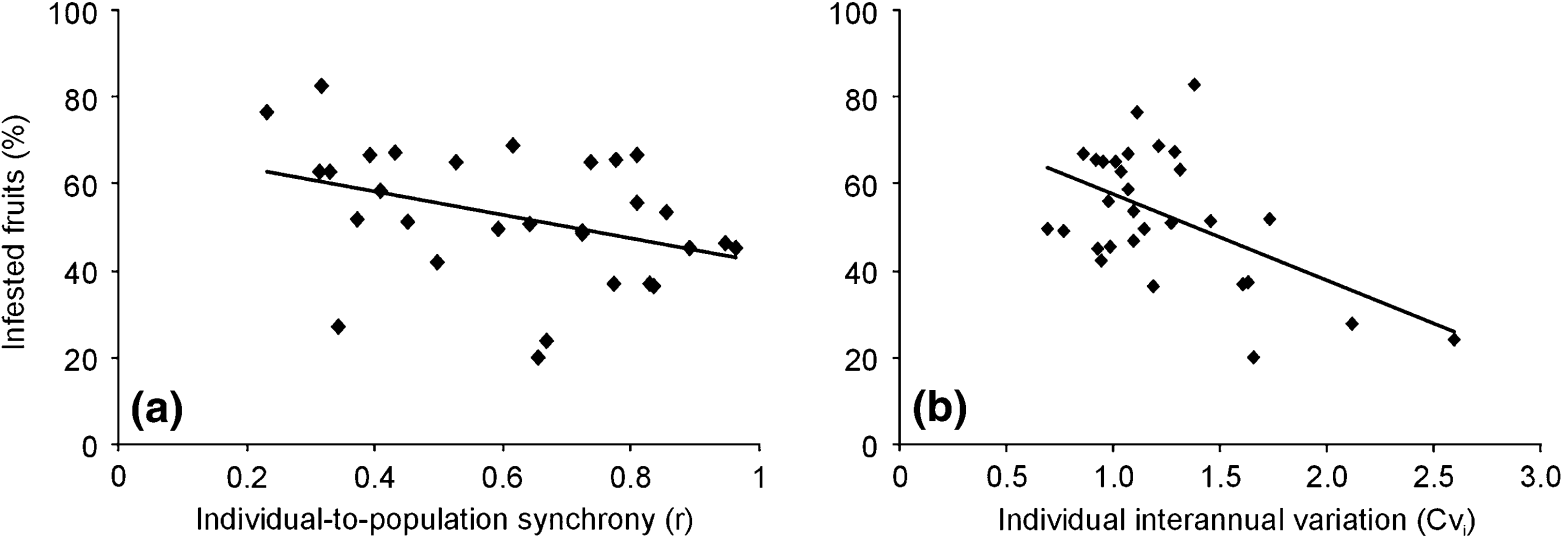
Infested fruits (%) as a function of **a** individual-to-population synchrony levels (measured as coefficients of the correlation between population and individual fruit production) and **b** individual interannual variation of fruit production (CV_*i*_)

The individual intensity of fruit infestation (percentage of infested fruits in 2005–2011) was related to the level of synchrony of fruit production between individual trees and the population. The highest was the level of synchrony the lowest was the percentage of infested fruits (*R* = −0.38; *P* = 0.04; Fig. 3a).

The individual intensity of fruit infestation was also negatively related with the individual variation of fruit production (CV_*i*_) (*R* = −0.53; *P* = 0.003; Fig. 3b).

## Discussion

Overall, pre-dispersal predation of rowan seeds by *A. conjugella* was high in most studied years. On average, it was 70 %. This is high in comparison with other species, most of which tend to have relatively low overall predation (Kolb et al. 2007). Fruit infestation was high even in some relatively heavy crop years. Seed loss in the population was probably considerable, not only for the seven studied years but also for the 12-year period of 2000–2011, as in the 5 years preceding the study of pre-dispersal seed predation (2000–2004), fruit production was low and without a year which could satiate predators (Żywiec et al. 2012). Seed predator pressure on the population is apparently very high.

We found both functional and numerical responses of predators to the variation of fruit production at the population level. Fruit infestation decreased with increasing fruit crop (functional response) and with increasing current- to previous-year fruit crop ratio (numerical response). The numerical response appears to be stronger. The importance of the relative size of current and previous year fruit crops on fruit infestation was especially conspicuous in 2006 and 2007, the second and third of three consecutive years of relatively heavy crops, with a considerable rise in the number of infested fruits. It indicated that the *A. conjugella* population was able to boost its density in consecutive years of high fruit production. Kelly (1994) suggested that predation pressure is an important selective force in such a situation. Our results confirm the notion that mast years should be separated by low crop years to reduce the impact of seed predators, an assumption of the predator satiation hypothesis (Janzen 1971; Silvertown 1980). Our population did not fulfill this condition and in consequence lost most of its fruit production in two relatively heavy crop years not preceded by low crop years. Consecutive mast years marked by significant increases of predation have been reported in other masting species (Hosaka et al. 2011).

Though we found both functional and quantitative responses of predators to the variation of fruit production, seed predation levels were significant even in heavy production years that followed low production years. The year of the heaviest fruit crop of the entire studied period, 2009, followed a year of almost no fruit production, although fruit predation was the lowest in the studied period, it still took a fifth of the fruit crop. This could be a result of prolonged diapause of *A. conjugella*, earlier found by Kobro et al. (2003). Extended diapause is a response of seed predators to unpredictable resource availability; it can make it very difficult for a plant to starve them (Kelly et al. 2000). In such a case, seed crops must be extremely variable across years if seed loss due to predation is to be reduced (Satake and Bjørnstad 2004). Such extremely high levels of masting in response to extended diapause were found in *Chinochloa* (CV_*p*_ range: 1.42–3.02; Kelly et al. 2000). In our population, the variation of seed crops was not very high (CV_*p*_ = 1.07; Żywiec et al. 2012), and it did not sufficiently reduce the *A. conjugella* population. For example, the relatively heavy crop year of 2005 followed five low crop years, and in that year, a 10-fold increase in fruit production was accompanied by nearly 40 % fruit infestation. These results show that when the ratio of current to previous year seed production is not high enough, predators may cope quite well with the increase in seed production. Similar findings were reported by Yasaka et al. (2003) and Kon et al. (2005). Our results suggest that effective reduction of fruit predation in the studied population requires periods of low fruit production separating heavy crop years longer than 1 year, and higher variation of fruit crops.

The predator satiation hypothesis states that predator pressure favours masting if the variation of seed crops satiates seed predators in heavy crop years (Janzen 1971; Silvertown 1980; Kelly 1994). In spite of substantial fruit infestation in all years, the functional and numerical responses of predators to the variation of fruit production were present in our study system. It implies that, under pressure from seed predators, the rowan trees preferred by selection should be those with higher variation of fruit production and higher synchronisation with the population. These individuals should have higher reproductive success, measured in our work as the percentage of uninfested fruits over 7 years. Indeed, we found that individuals which were expected to be preferred under seed predator pressure had higher reproductive success. With increasing synchrony of fruit production between individual trees and the population, the percentage of infested fruits decreased. There was also a negative relationship between the interannual variation of individual fruit production and the percentage of infested fruits. This confirms selection for individuals with a masting strategy.

Although we have found selection for masting individuals, the population does not seem well adapted to strong seed predation pressure. In the studied period, there were three consecutive years of relatively heavy crops in the population. The predator satiation hypothesis predicts that this should be strongly selected against (Norton and Kelly 1988). Moreover, in a previous study (Żywiec et al. 2012), we found a low level of synchronisation among individuals in heavy production years and moderate interannual variability of fruit production, which even fit within the range reported by Kelly et al. (2000) for plants that do not derive any selective benefits or disadvantages from mast seeding. Our study showed that the relationship between the reproductive success of individuals and their synchrony with the population was not very strong. At least three possible explanations for our findings can be proposed. The first relies on Lalonde and Roitberg’s (1992) suggestion that evolutionary interaction between masting and non-masting individuals is frequency-dependent, and that masting trees achieve the highest success when they constitute a large proportion of the population. If most individuals have synchronously heavy crop years, an individual out of synchrony loses its reproductive effort when seed predation and consequently the risk of being eliminated are high (Janzen 1971; Silvertown 1980). On the other hand, if the frequency of out-of-synchrony individuals is high, they reduce the effect of the quantitative response of predators by supplying resources to the predator population and maintaining it at high numbers (Lalonde and Roitberg 1992). The disadvantageous effects of unsynchronised individuals affect not only individuals representing that pattern but also synchronised individuals well fitted to seed predation, and this might weaken selection of better-fitted individuals. In the studied population, only a small group (21 %) of individuals showed the same pattern of heavy crop years as recorded for the whole population (Żywiec et al. 2012). Hence, our results confirm the suggestion of Lalonde and Roitberg (1992) that, in a population where any reproductive pattern dominates, selection for individuals best fitted to seed predation pressure can be difficult. The second possible explanation takes into account prolonged diapause of *A. conjugella*. The diapause model of Satake and Bjørnstad (2004) showed that, if a seed predator has extended diapause, plants suffer from severe seed losses over a large range of seed production patterns. The third possibility is related to the fact that rowan is pollinated by insects and dispersed by birds. Thus, fruit and seed production are under conflicting pressures: starvation and satiation of seed predators on the one hand, and avoidance of satiation and starvation of pollinators and dispersers on the other hand (Kelly 1994; Herrera et al. 1998; Koenig et al. 2003).

In the previous study, we found that fruit production of rowan at the population level was highly correlated with the weather conditions in the current and previous years (Żywiec et al. 2012). The present results demonstrate that individuals better synchronised with the population and individuals with higher interannual variability of fruit crops have higher reproductive success. A finding of switching in heavy crop years, that is, diversion of resources away from vegetative growth, would be evidence that variability of rowan fruit crops is also the effect of selection (Norton and Kelly 1988).

To conclude, our results showed that in a plant population experiencing a high level of seed predation and functional and quantitative responses of predators to the variation of fruit production, individuals expected to be preferred under seed predator pressure (i.e. with higher synchrony with the population and higher variation of fruit production) have higher reproductive success. Also, our data showed that, even when selection operates for masting individuals, the population can be weakly prepared for seed predation pressure. In the studied population, the relationship between plant and seed predator seems strongly affected by prolonged diapause of *A. conjugella*, which seems to give the predator a substantial advantage.
